# Mechanism of High-Fat Diet Regulating Rabbit Meat Quality Through Gut Microbiota/Gene Axis

**DOI:** 10.3390/ani15243608

**Published:** 2025-12-15

**Authors:** Gang Luo, Tongtong Xue, Kun Du, Zhanjun Ren, Yongzhen Luo

**Affiliations:** 1 College of Animal Science and Technology, Fujian Agriculture and Forestry University, Fuzhou 350007, China; 2 Animal Disease Prevention and Control and Healthy Breeding Engineering Technology Research Centre, Mianyang Normal University, Mianyang 621000, China; 3 College of Animal Science and Technology, Northwest A&F University, Xianyang 712100, China

**Keywords:** rabbit, biodiversity, jejunum, meat quality, IMF

## Abstract

With the improvement of living standards, people’s awareness of health care is becoming stronger and stronger. Rabbit meat is a very high-quality and healthy meat, but its consumption is low due to its poor flavor. To explore the regulatory mechanism of nutrition on the meat quality of rabbits, twenty-four rabbits were fed a control diet or a high-fat (5 percent lard) diet over 15 days. The contents and tissues of the jejunum were subjected to 16S sequencing and mRNA transcriptome sequencing, respectively. The results indicated that there were significant differences in species diversity through beta diversity analysis (*p* < 0.05). The diversity of alpha in the experimental group was also significantly reduced (*p* < 0.05). Based on gene function annotation, 8 bacteria at the phylum level and 11 bacteria at the genus level that are related to the metabolism of adipose tissue showed significant differences between the two groups (*p* < 0.05). The transcriptome results of the jejunum showed significant differences in 135 genes between the experimental group and control group (*p* < 0.05). Gene annotation revealed 10 differentially expressed genes related to fat metabolism, which regulate 36 signaling pathways. We speculated that *Alloprevotella* may influence drip loss and cooked meat rate by changing the expression of *PHGDH* through correlation analysis. In addition, *Coprococcus* may influence IMF by changing the expression of *NEDD4*, *ANGPTL3*, and *CYP8B1*. These results indicated that a high-fat diet changed the species and composition of bacteria in the rabbit jejunum. *Alloprevotella* and *Coprococcus* may influence rabbit meat quality and flavor by changing *PHGDH*, *NEDD4*, *ANGPTL3*, and *CYP8B1* gene expression in the host. This study laid a molecular foundation for the improvement of rabbit meat quality through nutritional diets.

## Simple Summary

With the outbreak of the COVID-19 pandemic, people’s awareness of health care is becoming stronger and stronger. Rabbit meat is known as a lean protein source that may have potential health benefits in light of its low fat and cholesterol content. Over the years, the consumption level of rabbit meat has remained very low. The main reason is that the taste and flavor of rabbit meat are not very good. The most important factors affecting rabbit meat are IMF and other meat quality indicators. We added 5% lard to the diet to explore the regulatory mechanism of fat deposition in rabbits. The jejunum of rabbits is the main organ for the digestion and absorption of fat. Therefore, we determined the species and number of microorganisms in the jejunum. In addition, we also examined the expression of genes in the jejunum. We found multiple signaling pathways regulating fat deposition. This study laid a molecular foundation for the improvement of rabbit meat quality by nutritional diets and may offer insights relevant to mammalian fat metabolism.

## 1. Introduction

Rabbit meat is a high-quality meat, which is rich in various beneficial elements that meet the needs of the human body [1,2,3]. However, low fat content affects the taste and leads to lower consumption levels. Studying the effects of HFDs on rabbits can help improve intramuscular fat deposition.

Fat is the most effective source of energy for animal life activities, which is essential for maintaining survival, growth and development, reproduction, and other functions. Studies show that an animal diet can affect intestinal barrier function [4]. HFDs can also increase levels of inflammatory factors *IL-1β* and *TNFα* in the intestinal epithelium [5,6]. When animals consume HFDs, the signaling pathways and gene expression that originally regulated fat metabolism will adapt to the HFD through changes. The above conclusions indicate that a high-fat diet can alter the expression of transcriptome genes in rabbits.

Rabbits are a type of low-fat animal that is highly sensitive to changes in body fat. The small intestine is an important digestive organ. The jejunum is the primary site of digestion and absorption in the small intestine. The jejunum has a lower pH value, higher oxygen content, and the presence of conjugated bile acids, providing different growth conditions for bacteria compared to other intestinal segments [7]. In addition, different nutrients (such as carbohydrates, proteins, fats, and trace elements) have the effect of regulating intestinal microbes, which, in turn, affects the health and metabolism of the body [8,9,10]. HFDs can significantly alter the composition of gut microbiota [11]. There are huge and complex microbiota in the gut of monogastric animals, which co-evolve with the host and participate in a variety of metabolic pathways and physiological processes. Therefore, it is also known as another important “organ” of the animal body [12], which plays an important role in the growth, development, and disease prevention of animals.

In this study, we explored the effect of HFD on the microbes and gene expression in the jejunum of rabbits. Further, it is speculated that the specific mechanism of dietary fat regulates meat quality in rabbits. The results showed that HFDs not only affected the population and composition of many intestinal microorganisms but also caused great changes in the expression of jejunal genes. Through correlation analysis, we linked the *Alloprevotella* and *PHGDH* gene with meat quality indicators (drip loss and cooked meat rate). In addition, we also found a correlation among *Coprococcus*, genes (*NEDD4*, *ANGPTL3*, and *CYP8B1*), and IMF. This study laid a molecular foundation for the improvement of rabbit meat quality through nutritional diets.

## 2. Materials and Methods

### 2.1. Animals

Twenty-four Igel rabbits (About 450 g), 21 days after birth, were selected as experimental animals, whose weight, breed, physiological status, etc., were basically the same. The power calculation results showed that the power value was greater than 0.8 (https://tooldone.com/zh/tongji/gonglu-fenxi-jisuanqi/) (accessed on 5 August 2025). All experimental animals were randomly divided into a control group and an experimental group. Animals in the control group (full diet) and experimental group (full diet + 5% lard) were fed for 15 days after a 2-day adaptation period. All rabbits used in the experiment were free-feeding and were raised in the same environment.

### 2.2. Sample Collection

Six rabbits from each group were selected for further trials at the end of the feeding trial. Rabbits were sacrificed by injection of 100 mg/kg of pentobarbital sodium. The jejunum was rapidly isolated after the rabbits were slaughtered. Then, samples of jejunal contents and jejunal tissues were collected separately in 2 mL cryopreservation tubes. The samples were frozen in liquid nitrogen and stored in a −80 °C freezer.

### 2.3. Experimental Library Construction and Sequence

The contents of the jejunum were used to extract total DNA by using PowerSoil^®^ DNA Isolation Kit (Tsingke Biotechnology Co., Ltd., Beijing, China). A two-step library-building method was used to build a microbial diversity library after amplification of primers for different microbial species. In the first step, DNA was used as a template to design primers with adapters for PCR. In the second step, PCR was performed using the first step product as a template. The specific process includes target region PCR, Solexa PCR, quantification and sample mixing, purification after sample mixing, gel cutting recovery, and on-line sequencing. The constructed library was sequenced using Illumina novaseq6000 PE250 (Beijing Dequan Xingye Trading Co., Ltd., Beijing, China). Jejunum tissue is used to extract total RNA. The concentration of extracted RNA was detected using nanodrop2000 (Thermo Fisher Scientific, Waltham, MA, USA), and Agient2100 and LabChip GX (Agilent Technologies, Santa Clara, CA, USA) were used to detect the integrity. MRNA was used to build the library, and the specific method includes 9 steps. MRNA was purified using magnetic beads and was interrupted as required in a PCR instrument. The interrupted RNA was reverse transcribed to synthesize the first and second strands of cDNA at one time. The product was end-repaired and connected to the connector. Purification and library quality inspection were performed after PCR amplification. Finally, a Huada DNBSEQ-T7 gene sequencer (Shenzhen Huada Intelligent Manufacturing Technology Co., Ltd., Shenzhen, China) was used for sequencing.

### 2.4. Quality Control

Microbial sequencing results were filtered using Trimomatic v0.33 software. Clean reads were obtained after identifying and removing primer sequences using the cutadapt 1.9.1 software. Finally, the valid data was obtained by denoising using dada2 [13] from QIIME2 (2021.2) [14]. The raw image data files obtained from high-throughput sequencing are converted into sequencing raw data through CASAVA base recognition analysis. The raw data was processed using fastp-V0.20.1 (https://github.com/OpenGene/fastp, accessed on 5 August 2025) software to remove joints and low-quality sequences, resulting in clean data.

### 2.5. Statistical Analysis

We used the RDP (FDR correction was used) classifier algorithm to perform classification analysis on representative sequences of operational classification units with a similarity level of 97.0% [15,16]. Diversity analysis of α and β was performed using mothur [17]. LEfSe [18] analysis was used to identify species with significant differences. HISAT2-2.0.4 (https://daehwankimlab.github.io/hisat2/, accessed on 5 August 2025) was used to align clean data with the genome. The assembly and merging of genes or transcripts were completed by StringTie-1.3.4d (http://ccb.jhu.edu/software/stringtie/, accessed on 5 August 2025) software. Transcript and annotation results were detected using gffcompare-0.9.8 (http://ccb.jhu.edu/software/stringtie/gffcompare.shtml, accessed on 5 August 2025) software. We used the ballgown package to provide file input for FPKM quantification. Then, DEseq2-3.22 (https://bioconductor.org/news/bioc_3_22_release/, accessed on 5 August 2025) was used to analyze the differences between samples, and genes with a fold change >2 and *p*-value < 0.01 were defined as differentially expressed genes.

## 3. Results

### 3.1. Summary of 16S Sequencing Data

**Figure 1:**
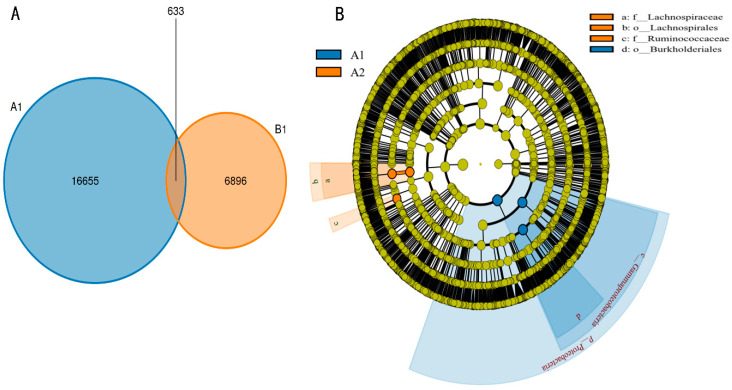
(**A**) Differences and similarities of ASV in different groups; (**B**) analysis of the evolutionary branch diagram of LEfSe.

As shown in Table 1, we have counted the number of different samples at the levels of kingdom, phylum, class, order, family, genus, and species. The results of Figure 1A indicated that there were 16,655 ASV in the control group, 6896 ASV in the experimental group, and 633 common ASV in the two groups. In addition, we found significant differences in bacteria between the control group and the experimental group at different levels (Figure 1B).

### 3.2. Diversity of Bacteria

**Figure 2:**
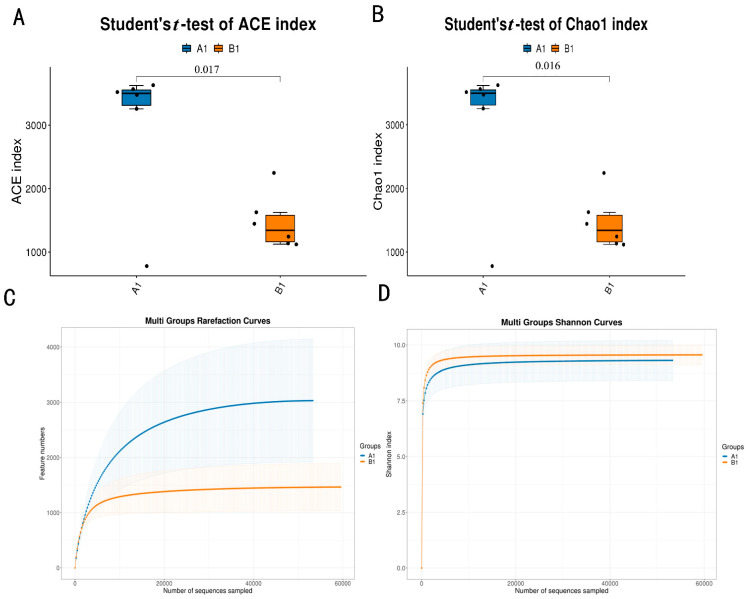
Alpha diversity analysis. (**A**) Alpha diversity intergroup differences in ACE index; (**B**) alpha diversity intergroup differences in Chao1 index; (**C**) species diversity (rarefaction curve); (**D**) species diversity (Shannon Index).

**Figure 3:**
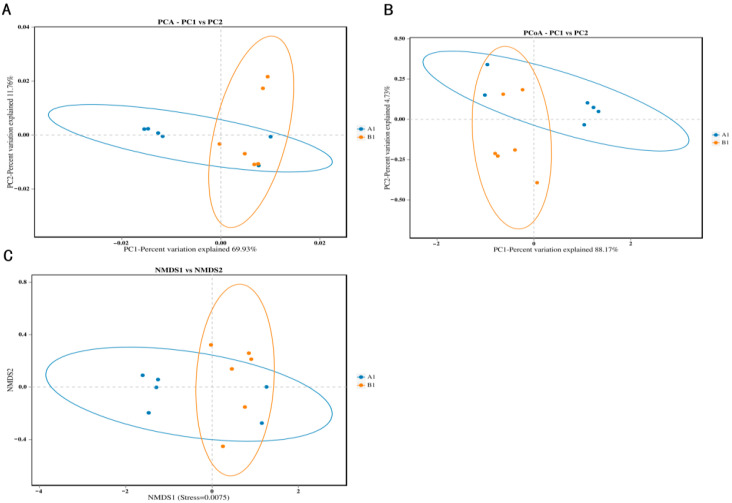
Beta diversity analysis. (**A**) PCA analysis; (**B**) PCoA analysis; (**C**) NMDS analysis.

In the results of the α diversity analysis, we found that the ACE index and chao1 index of the control group were significantly higher than those of the experimental group (Figure 2A,B) (*p* < 0.01). The results of the rarefaction curve indicated that a large number of species were found in the control group community compared to the experimental group during sequencing (Figure 2C). The Shannon index results also indicated that the control group has more species and a wider variety of species compared to the experimental group (Figure 2D). The analysis of β diversity is mainly completed through three methods: PCA, PCoA, and NMDS. The results of PCA, PCoA, and NMDS indicated that the similarity and difference within the group are small, while the similarity and difference between groups are large (Figure 3A–C).

### 3.3. Bacteria Related to Lipid Metabolism at the Genus Level

**Figure 4:**
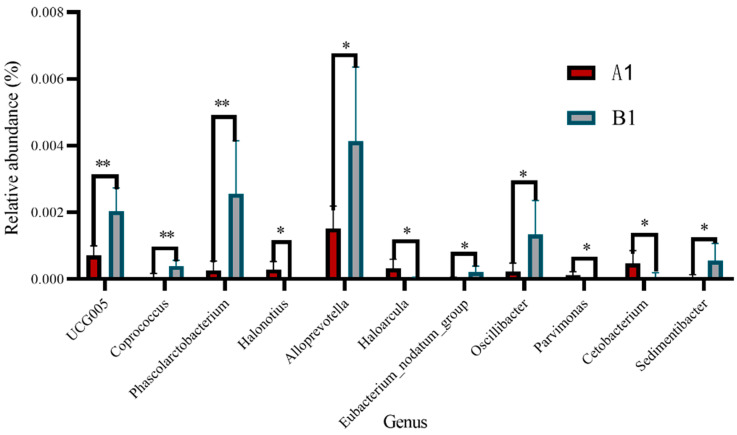
Inter-group differences in abundance related to fat metabolism in genus (*, *p* ≤ 0.05; **, *p* ≤ 0.01).

Based on functional annotations, there were significant differences in 11 bacteria related to fat metabolism between the control group and the experimental group. The abundance of *Halonotius*, *Haloarcula*, *Parvimonas*, and *Cetobacterium* was significantly higher in the control group than in the experimental group (Figure 4) (*p* < 0.05). The abundance of *UCG005*, *Coprococcus*, and *Phascolarctobacterium* was extremely significantly lower in the control group than in the experimental group (Figure 4) (*p* < 0.01). In addition, the abundance of *Alloprevotella*, *Eubacterium-nodatum-group*, *Oscillibacter*, and *Sedimentibacter* also significantly increased in the experimental group (Figure 4) (*p* < 0.05).

### 3.4. Functional Distribution of Differentially Expressed Genes

**Figure 5:**
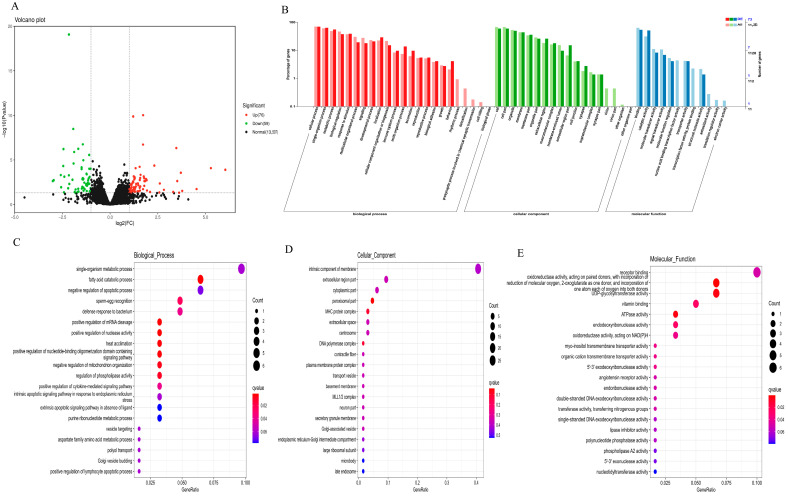
Functional distribution of differential genes. (**A**) Differential expression (volcano plot); (**B**) differential expression gene GO annotation classification statistics; (**C**) gene ratio in biological process; (**D**) gene ratio in cellular component; (**E**) gene ratio in molecular function.

As shown in Figure 5A, we found a total of 135 differential genes in the transcriptome sequencing of the rabbit jejunum. Compared to the control group, the experimental group had 76 genes upregulated and 59 genes downregulated. The results of functional annotation indicated that differentially expressed genes mainly regulate molecular functions, cellular components, and biological processes (Figure 5B). Gene ratios involved in molecular functions, cellular components, and biological processes were shown in Figure 5C–E. Among them, differentially expressed genes related to fat metabolism account for a large proportion of molecular functions and cellular components.

### 3.5. The Regulatory Mechanism of Differentially Expressed Genes Related to Fat Metabolism

**Figure 6:**
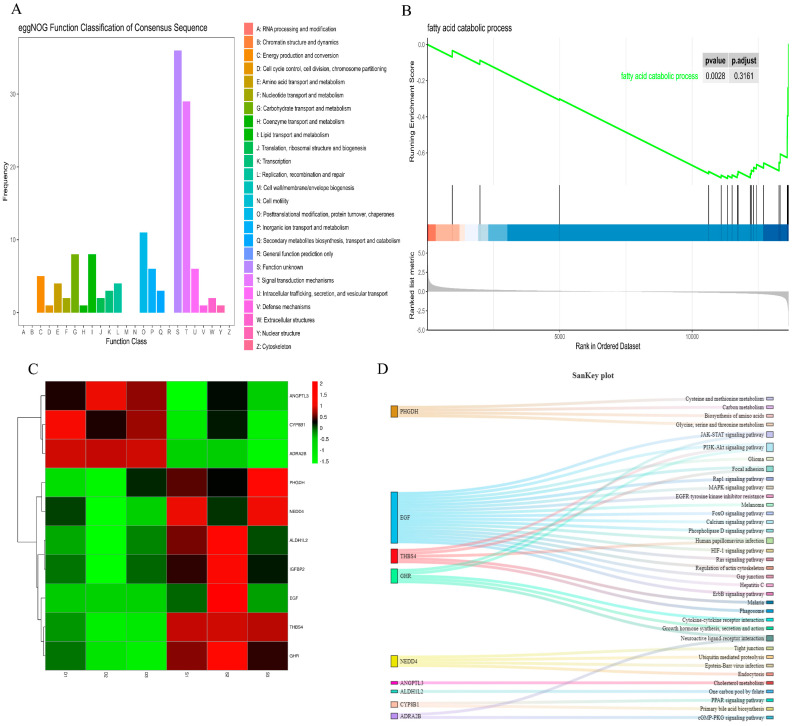
(**A**) Classification and statistics of differentially expressed genes by eggNOG annotation; (**B**) gene set enrichment analysis of fatty acid catabolic process, The abscissa represents the position information of the sorted gene set, and the black vertical line in the abscissa represents the genes in the GO Term/KEGG pathway; (**C**) differential gene heatmap related to fat metabolism; (**D**) Sankey diagram of differentially expressed genes and signaling pathways related to fat metabolism.

Differential gene COG (Cluster of Orthologous Groups of proteins) classification found that the number of genes regulating lipid transport and metabolism processes ranked fourth (Figure 6A). The gene set enrichment analysis of the fatty acid decomposition metabolism process also showed significant differences between the control group and the experimental group (Figure 6B) (*p* < 0.01). As shown in Figure 6C, there were significant differences in 10 genes related to fat deposition between the control group and the experimental group (*p* < 0.01). In addition, we found that nine genes regulate fat metabolism in 36 signaling pathways through different pathways (Figure 6D).

### 3.6. Regulation Mechanism of Microorganisms on the Meat Quality of Rabbits by Affecting Host Genes

**Figure 7:**
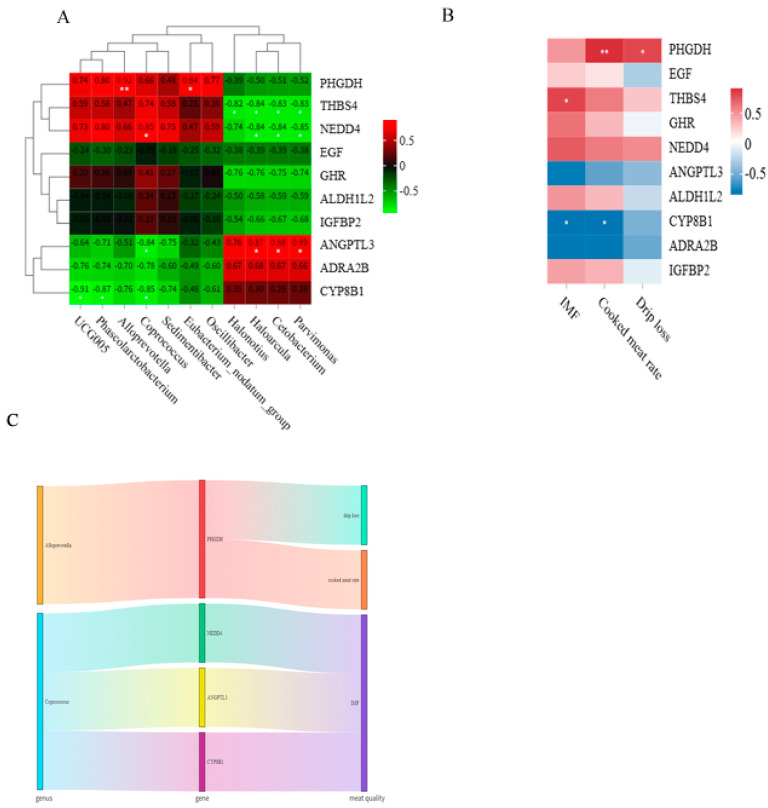
(**A**) Screening the correlation heatmap between genus and genes; (**B**) correlation heatmap between meat quality and genes; (**C**) Sankey diagram of genus, genes, and meat quality; (*, *p* ≤ 0.05; **, *p* ≤ 0.01).

In order to explore the relationship between genes related to fat deposition and bacteria, we conducted a correlation analysis between genes and bacteria. As shown in Figure 7A, expression of *PHGDH* was extremely significantly positively correlated with the abundance of *Alloprevotella* (*p* < 0.01). *PHGDH* expression was significantly positively correlated with *Eubacterium_nodatum_group* abundance (*p* < 0.05). The expression of *THBS4* was negatively correlated with the abundance of *Halonotius*, *Haloarcula*, *Parvimonas*, and *Cetobacterium* (*p* < 0.05). *NEDD4* expression was negatively correlated with the abundance of *Haloarcula*, *Parvimonas*, and *Cetobacterium*, but positively correlated with the abundance of *Coprococcus* (*p* < 0.05). Conversely, *ANGPTL3* expression was positively correlated with the abundance of *Haloarcula*, *Parvimonas*, and *Cetobacterium*, but negatively correlated with the abundance of *Coprococcus* (*p* < 0.05). In addition, the expression of *CYP8B1* was negatively correlated with the abundance of *Coprococcus*, *UCG005*, and *Phascolarctobacterium* (*p* < 0.05). In addition, we found that IMF was positively correlated with the expression of the *THBS4* gene (*p* < 0.05) (Figure 7B). *CYP8B1* expression was negatively correlated with IMF and cooked meat rate (*p* < 0.05) (Figure 7B). *PHGDH* expression was positively correlated with cooked meat rate and drip loss (*p* < 0.05) (Figure 7B). Through correlation analysis, we speculated that *Alloprevotella* regulates drip loss and cooked meat rate by affecting the expression of *PHGDH* (Figure 7C). In addition, *Coprococcus* regulated IMF by affecting the expression of *NEDD4*, *ANGPTL3*, and *CYP8B1* (Figure 7C). The specific regulatory mechanism is still being further verified.

## 4. Discussion

Rabbit meat is a kind of healthy meat suitable for human consumption. However, the meat quality and flavor seriously affect the consumption of rabbit meat. One of the main factors that determines the meat quality and flavor of rabbit meat is the deposition of fat. The jejunum is the main organ for animal digestion and absorption of fat, and bacteria composition and the expression of genes are important factors in regulating animal fat. The metabolism of gut microbiota is closely related to the diet and evolution of the host system [19,20]. This study found that the diversity of contents in the jejunum of rabbits significantly decreased after consuming a HFD, indicating that a HFD can inhibit the types and quantities of bacteria. Studies have also shown that rabbits fed a high-fat diet can reduce the abundance of bacteria in the cecum [21]. These results indicate that a HFD reduces the abundance and diversity of bacteria.

In order to further reveal the effects of HFDs on lipid metabolism-related bacteria, we conducted further analysis at the genus level. We found *UCG005*, *Coprococcus*, *Alloprevotella*, *Eubacterium_nodatum_group*, *Oscillibacter*, *Sedimentibacter*, and *Phascolarctobacterium* increased after feeding rabbits a high-fat diet. *UCG005* is a potential biomarker closely related to oxidative stress and metabolic genes [22]. Studies have also found that reducing fat deposition reduced the abundance of *UCG005*, *Alloprevotella*, and *Oscillibacter* in mice [23,24]. The abundance of *Coprococcus* and *Eubacterium_nodatum_group* increased when feeding cows HFDs [25,26]. *Sedimentibacter* can degrade long-chain fatty acids and function in high-lipid environments [27]. *Phascolarctobacterium* can produce short-chain fatty acids [28]. These results are consistent with the findings of this study. In addition, the abundance of *Halonotius*, *Haloarcula*, *Parvimonas*, and *Cetobacterium* decreased after feeding rabbits a HFD. The enrichment of *Halonotius* and *Haloarcula* is closely related to salt [29,30]. Reducing fat deposition in zebrafish can upregulate the abundance of *Cetobacterium* [31]. *Parvimonas* has a significant impact on inflammation in cattle [32]. In summary, we speculated that HFDs may regulate the mechanism of fat deposition in rabbits by affecting the types and composition of bacteria.

To further explore the effects of HFDs on fat absorption and digestion in rabbits, we detected the expression of the rabbit jejunal transcriptome. The results indicated that a large number of genes had undergone changes in expression, which suggested that HFDs can alter gene expression. Functional annotation revealed that these genes played important roles in lipid metabolism, growth, and development, as well as in immunity. As shown in Figure 6A,B, a large proportion of differentially expressed genes played a role in lipid metabolism and fatty acid processes, indicating that they may regulate fat deposition. Screening revealed 10 genes regulating fat deposition. *ANGPTL3*, *CYP8B1*, and *ADRA2B* expression decreased after feeding rabbits a high-fat diet. Studies have found that knocking out the *ANGPTL3* gene can induce fat production [33]. *CYP8B1* played an important role in animal weight gain and fat absorption [34]. Inhibiting fat production in mice can lead to an increase in the expression of *ADRA2B* [35]. The above conclusion was consistent with the results of this study, indicating that an increase in body fat will reduce the expression of *ANGPTL3*, *CYP8B1*, and *ADRA2B*. Conversely, the expression of *PHGDH*, *EGF*, *THBS4*, *GHR*, *NEDD4*, *ALDH1L2*, and *IGFBP2* increased after feeding rabbits a high-fat diet. *PHGDH* [36], *EGF* [37], *THBS4* [38], *GHR* [39], *NEDD4* [40], and *ALDH1L2* [41] can promote fat deposition or high expression in adipose tissue. In addition, nine differentially expressed genes regulated 36 signaling pathways through multiple pathways. Functional annotation revealed that these signaling pathways played an important role in the process of fat deposition. In summary, we speculated that changes in gut microbes were linked to changes in host gene expression.

To explore the relationship between bacteria and key genes involved in fat deposition, we conducted a correlation analysis. As shown in Figure 7, we speculated that Alloprevotella regulated drip loss and cooked meat rate by affecting the expression of *PHGDH*. In addition, *Coprococcus* regulated IMF by affecting the expression of *NEDD4*, *ANGPTL3*, and *CYP8B1*. Studies have shown that gut microbiota and host genes can regulate animal physiological mechanisms through interactions [42,43]. This result is consistent with our speculation that gut microbes affect host gene expression. Studies have shown that the *PHGDH* gene can change the proliferation and development of muscle cells [44]. So, *PHGDH* can regulate drip loss and cooked meat rate by changing muscle structure. Previous studies have shown that *NEDD4* [45], *ANGPTL3* [46], and *CYP8B1* [47] genes regulate fat deposition in different tissues. The above results confirmed our speculation that *Coprococcus* regulates IMF by affecting the expression of *NEDD4*, *ANGPTL3*, and *CYP8B1*.

## 5. Conclusions

In summary, we revealed that a high-fat diet reduced microbial diversity in the jejunum of rabbits and altered gene expression in the jejunum. High-fat diets increased the abundance of seven bacteria and decreased the abundance of four bacteria at the genus level, all of which played important roles in fat metabolism. In addition, a high-fat diet promoted the expression of seven genes and inhibited the expression of three genes. *Alloprevotella* regulated drip loss and cooked meat rate by affecting the expression of *PHGDH*. In addition, *Coprococcus* regulated IMF by affecting the expression of *NEDD4*, *ANGPTL3*, and *CYP8B1*. These findings show that microbe–gene interactions may affect fat metabolism and meat quality in rabbits.

## Figures and Tables

**Table 1:** The number of different levels of microbial communities.

Sample	Kingdom	Phylum	Order	Family	Genus	Species
L35D1	2	42	247	455	860	1490
L35D2	2	34	193	338	608	981
L35D3	2	43	259	473	884	1467
L35D4	2	42	246	456	844	1414
L35D5	2	26	131	210	317	393
L35D6	2	41	250	438	845	1449
L35S1	2	41	220	383	691	1041
L35S2	2	36	180	293	485	635
L35S3	2	28	162	264	418	575
L35S4	2	33	181	307	506	730
L35S5	2	36	176	286	480	632
L35S6	2	37	194	316	551	766

## Data Availability

Raw data can be obtained from the NCBI database. The submission ID of the microorganism is SUB15458652, and the BioProject ID is PRJNA1291025. The submission ID of the transcriptome is SUB15461282.

## Abbreviations

HFD: High-fat diets; PHGDH: phosphoglycerate dehydrogenase; EGF: Epidermal Growth Factor; THBS4: Thrombospondin 4; GHR: growth hormone receptor; NEDD4: neural precursor cell expressed, developmentally downregulated protein 4; ANGPTL3: Angiopoietin-Like Protein 3; ALDH1L2: Aldehyde Dehydrogenase 1 Family Member L2; CYP8B1: cytochrome P450 family 8 subfamily B member 1 Gene; ADRA2B: Adrenoceptor Alpha 2B; IGFBP2: insulin like growth factor binding protein 2.
